# Limonene nanoemulsified with soya lecithin reduces the intensity of non-isothermal treatments for inactivation of *Listeria monocytogenes*

**DOI:** 10.1038/s41598-020-60571-9

**Published:** 2020-02-27

**Authors:** Alberto Garre, Jennifer F. Espín, Juan-Pablo Huertas, Paula M. Periago, Alfredo Palop

**Affiliations:** 1 Food Microbiology, Wageningen University & Research, P.O. Box 17, 6700 AA Wageningen, The Netherlands; 2 Dpto. Ingeniería Agronómica, Instituto de Biotecnología Vegetal, Campus de Excelencia Internacional Regional “Campus Mare Nostrum”, Escuela Técnica Superior de Ingeniería Agronómica, Universidad Politécnica de Cartagena, Cartagena, Spain

**Keywords:** Antimicrobials, Applied microbiology

## Abstract

Consumers’ demands for ready-to-eat, fresh-like products are on the rise during the last years. This type of products have minimal processing conditions that can enable the survival and replication of pathogenic microorganisms. Among them, *Listeria monocytogenes* is of special concern, due to its relatively high mortality rate and its ability to replicate under refrigeration conditions. Previous research works have shown that nanoemulsified essential oils in combination with thermal treatments are effective for inactivating *L. monocytogenes*. However, previous research works were limited to isothermal conditions, whereas actual processing conditions in industry are dynamic. Under dynamic conditions, microorganism can respond unexpectedly to the thermal stress (e.g. adaptation, acclimation or increased sensitivity). In this work, we assess the combination of nanoemulsified *D*-limonene with thermal treatments under isothermal and dynamic conditions. The nanoemulsion was prepared following an innovative methodology using soya lecithin, a natural compound as well as the essential oil. Under isothermal heating conditions, the addition of the antimicrobial enables a reduction of the treatment time by a factor of 25. For time-varying treatments, dynamic effects were relevant. Treatments with a high heating rate (20 °C/min) are more effective than those with a slow heating rate (1 °C/min). This investigation demonstrates that the addition of nanoemulsified *D*-limonene can greatly reduce the intensity of the thermal treatments currently applied in the food industry. Hence, it can improve the product quality without impacting its safety.

## Introduction

One of the present trends of citizens in developed countries is a higher demand for fresh-like products with nutritional and quality properties similar to raw products^1^. Linked to the increased consumption of these fresh-like foods, the incidence of foodborne illnesses associated to these food types has also grown. One of the foodborne pathogens of greatest concern in developed countries is *Listeria monocytogenes*^2^. This microorganism is the causative agent for the illness called listeriosis^3^, whose mortality rate is about 14%^2^ (one of the highest among the foodborne diseases). *L. monocytogenes* has been found in a wide variety of food products where it is able to grow at refrigeration temperatures (dairy, raw meat, raw vegetables or ready-to-eat foods)^4,5^. In spite of the efforts by regulatory agencies and governments, there has been a significant increase of the listeriosis cases in the European Union during the period 2008–2017^2^. One of the latest outbreaks of listeriosis has taken place in Spain involving more than 200 confirmed cases, as well as several deaths and miscarriages^6^. Besides being a hazard for public health, the contamination of food by this microorganism can cause irreparable damage to the image of a company^7^. *L. monocytogenes* has the ability to form biofilms and replicate in this state, increasing its potential to prevail in food industries^8^. Consequently, there is a need to find more effective treatments for inactivating this microorganism.

The application of heat treatments is the most common technology used in food industries to inactivate pathogenic microorganisms and render food products safe. However, the application of high temperatures can also have a negative impact on the nutritional and sensorial attributes of the food^9^. Due to consumers’ demands for high quality products, present food industries are pushed towards reducing the intensity of the treatments or seeking alternative technologies that can complement and/or substitute thermal treatments. Among these technologies, the use of antimicrobials has gained popularity during the last years^10^. Of special interest are compounds of natural origin with an antimicrobial effect, probably due to the consumers distrust for synthetic antimicrobials. Among these natural compounds, components of essential oils have shown remarkable antimicrobial and even antioxidant and anti-carcinogenic properties^11,12^. The antimicrobial action of essential oils has been attributed to their ability to interact with the membrane of microbial cells, which they can often penetrate. Then, they can cause the leakage of different elements of the cytoplasm, finally leading to cellular breakdown^13^.

However, there are still several technological challenges that limit the application of natural antimicrobials in food industries. They are susceptible to oxidative degradation (which greatly reduces their effect) and they have a pronounced aromatic character that can impact the flavor of the food product. Moreover, they are immiscible in aqueous media, which hampers their application in food products. Therefore, the development of technological solutions to optimize the application of these compounds in foods has become an important part of the research in food science. Nanoemulsion technology has emerged in the last years and is providing very promising results^14–16^. Nanoemulsions, with droplet size of up to 0,5 µm, are usually more kinetically stable than coarse emulsions^17^. This results in an increase in the presence of the antimicrobial agent in food matrices, where they may interact with the relevant foodborne microorganisms^18,19^. Nanoemulsions of essential oils have provided satisfactory results against a wide variety of microorganisms. Their antimicrobial effect was even more acute when applied in a nanoemulsified form^12,14,18,20–22^. The reason for that is that the smaller size of the nanuemulsified droplets increases the surface area coming in contact with bacterial cells and promotes the interaction between the antimicrobial agent and the cell membrane of the microorganism^22–24^.

Among the essential oils, *D-*limonene has shown remarkable antimicrobial properties when combined with thermal treatments^20,25–27^. *D-*limonene is an important flavour component in citrus essential oil (lemon, orange, tangerine, etc.), and has the generally regarded as safe (GRAS) status^28^. Maté *et al*.^25,26^ and Ros-Chumillas *et al*.^27^ have studied the synergistic effect of a mild heat treatment and an essential oil nanoemulsion. The synergistic effect led to a 100-fold reduction of the thermal resistance of *L. monocytogenes* both in culture medium^26^ and in apple juice^25^, and a 50-fold reduction of the thermal resistance of *Salmonella* Senftenberg^27^, which are, by far, the largest reductions in microbial heat resistance ever published when combining heat with natural antimicrobials. However, these studies were limited to the application of isothermal treatments (constant temperature), whereas in the ones actually applied in industry the temperature is dynamic. Indeed, to our knowledge, no previous research has been performed on the effect of such combined processes on dynamic thermal treatments. Although thermal inactivation of microbial cells has been studied for over a century, due to the limitations of the experimental equipment, until the late 1990s most studies were limited to isothermal conditions. In the last couple of decades, the study under controlled laboratory conditions of dynamic treatments has shown the difficulties for predicting the microbial response under dynamic conditions based on isothermal experiments^29^. There is empirical evidence that dynamic treatments may enable microbial cells to develop a physiological response that may increase their resistance to the thermal stress^30–34^. On the other hand, some research works have observed that a rapid heating of the cells may result in an increased sensitivity, reducing its resistance to posterior stresses^35^. These physiological mechanisms are not yet well understood at a molecular level and is currently an active field of research^36,37^. Consequently, it is required to perform dynamic experiments at a population level to observe possible interactions between the temperature level, the dynamics of temperature and other effects, such as the presence of an antimicrobial. Hence, the aim of this study is to evaluate the combined effect of a thermal treatment with a nanoemulsion of *D*-limonene, using an innovative emulsification technique based on soya lecithin, on the inactivation of *L. monocytogenes*. We have performed experiments under isothermal and dynamic heating conditions to assess the interaction between the dynamics of the temperature and the effect of the nanoemulsified natural antimicrobial.

## Results

**Figure 1:**
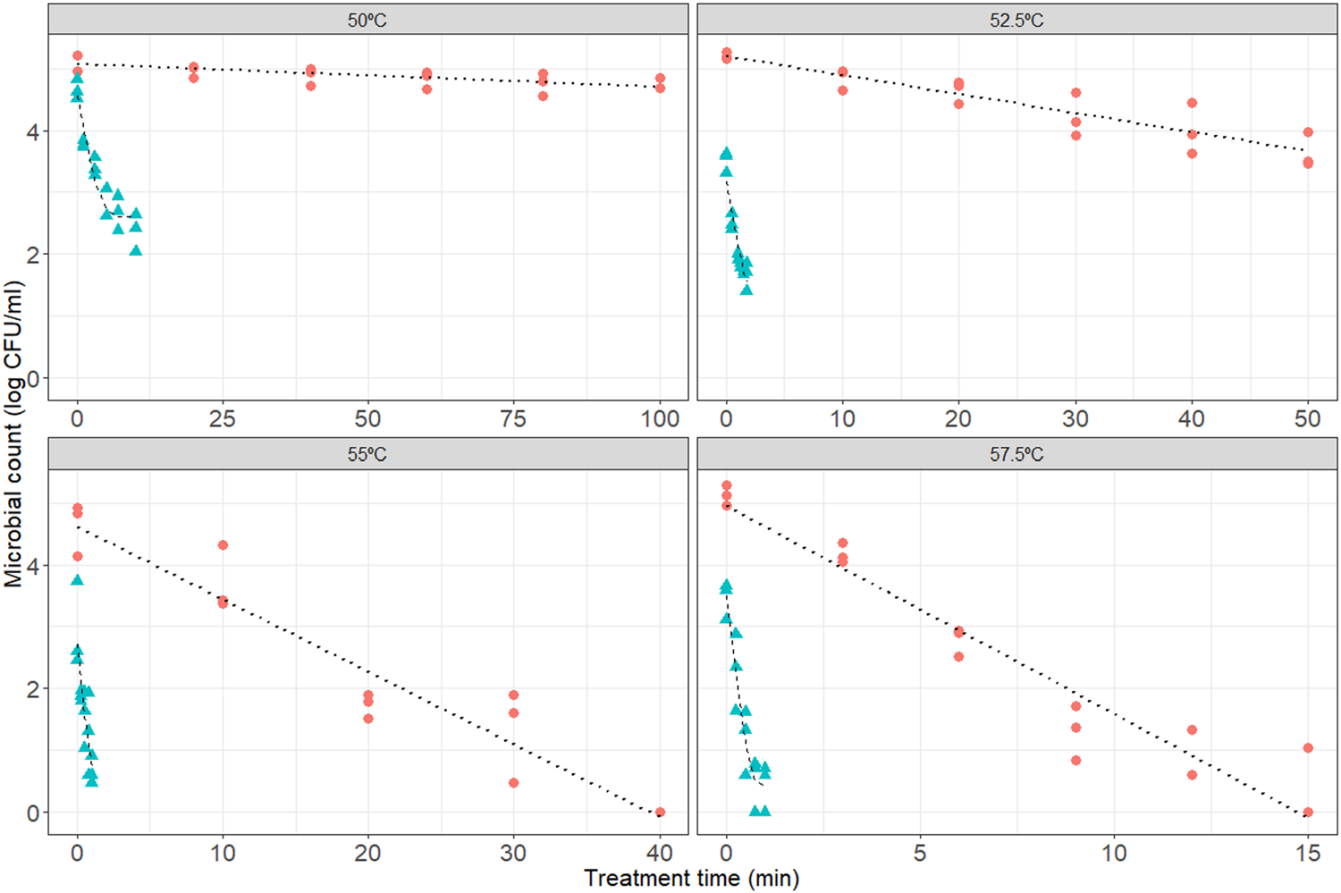
Survivor curves obtained under isothermal conditions. Red dots indicate treatments without the addition of the nanoemulsified limonene. Samples with limonene are shown are blue triangles. The lines illustrate the model fits (dashed for samples with limonene, dotted for samples without). The facets represent different treatment temperatures (note the different scales in the x-axis).

Figure 1 compares the survivor curves obtained under isothermal conditions for control samples (without nanoemulsified *D-*limonene added to the heating medium) and those with nanoemulsified *D*-limonene. The addition of the natural antimicrobial to the heating medium has a dramatic impact on the resistance of the *L. monocytogenes* cells to the treatment. At 50.0 °C, the control treatment was unable to reduce the microbial count. However, the treatment at the same temperature supplemented with nanoemulsified limonene reduced the microbial count by 3 log-cycles after only 15 minutes. This level of inactivation is similar to the one attained for the control treatment at 57.5 °C after the same treatment time (15 minutes). Consequently, the addition of the nanoemulsified natural antimicrobial enables to reduce the treatment temperature from 57.5 °C to 50.0 °C without a negative impact on the safety of the product with respect to *L. monocytogenes*.

The Geeraerd model was able to describe the microbial response in every case analysed (model predictions are illustrated in Fig. 1). This mathematical model enables to quantify the impact of the nanoemulsified limonene on the thermal resistance of the microbial cells. Table 1 reports the model parameters estimated for each condition. For the control samples, a D-value of 16.1 min (±0.9 min) was estimated at 53.8 °C. When the natural antimicrobial was added to the heating medium, the D-value was reduced by a factor of 25 (0.6 ± 0.1 min at 53.8 °C). Therefore, according to the model predictions, the addition of the nanoemulsified limonene would enable a reduction in the duration of the inactivation treatment at 53.8 °C by a factor of 25 without impacting the microbial safety of the product. For every treatment where the nanoemulsified limonene was added to the media, we observed a tail effect, whose height decreased with the treatment temperature (2.6 ± 0.1 log CFU/ml at 50.0 °C, 1.2 ± 0.6 log CFU/ml at 52.5 °C, 0.4 ± 0.5 log CFU/ml at 55.0 °C and 0.4 ± 0.2 log CFU/ml at 57.5 °C). Tail effects are common in microbial inactivation and can be attributed to several causes. They can be an indicator of a fraction of the microbial population that is more resistant to the treatment due to the inherent between-cell variability^38,39^. Another explanation for tail effects is that they are an artefact caused by the sampling error associated to the plating technique^38,40^. Garre *et al*.^40^, based on numerical simulations, provided upper bounds for the tail effects to be expected for different experimental settings. Accordingly, the tail effects observed at 55.0 and 57.5 °C could be artefacts, whereas those observed at 50.0 and 52.5 °C are representative of a resistant sub-population.

**Table 1:** Model parameters (±standard error) of the Geeraerd model estimated from isothermal inactivation experiments.

	Parameter	Value
Control samples	D-value at 53.75 °C	16.10 ± 0.85 min
	z-value	4.95 ± 0.18 °C
Samples with nanoemulsified limonene	D-value at 53.75 °C	0.64 ± 0.07 min
	z-value	7.45 ± 0.79 °C
	log *N*_*tail*_ at 50 °C	2.60 ± 0.14 log CFU/ml
	log *N*_*tail*_ at 52.5 °C	1.16 ± 0.61 log CFU/ml
	log *N*_*tail*_ at 55 °C	0.42 ± 0.48 log CFU/ml
	log *N*_*tail*_ at 57.5 °C	0.42 ± 0.17 log CFU/ml

**Figure 2:**
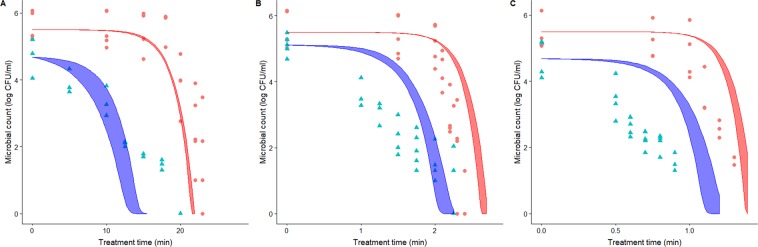
Comparison between predictions based on isothermal data and survivor curves for dynamic treatments with a heating rate of (**A**) 1 °C/min, (**B**) 10 °C/min and (**C**) 20 °C/min. Red dots indicate treatments without the addition of the nanoemulsified limonene. Samples with limonene are shown are blue triangles. The ribbons represent the prediction intervals (95% confidence) based on isothermal experiments for control samples (red) and samples with nanoemulsified limonene (blue).

Figure 2 compares the microbial counts of *L. monocytogenes* observed for control samples (red dots) and those supplemented with nanoemulsified *D-*limonene for non-isothermal treatments with different heating rates. As well as for the isothermal case, the addition of the nanoemulsified antimicrobial has a strong influence on the thermal resistant of the bacterial cells. For the treatment with a heating rate of 1 °C/min (Fig. 2A), the addition of the nanoemulsified antimicrobial reduces the time required to cause 3 log-reductions in the count of *L. monocytogenes* from 22 min to 12 min. Similarly, for the treatment with a heating rate of 10 °C/min, the time is reduced from 2.5 min to 1.5 min. Finally, for the treatment with a heating rate of 20 °C/min, the time to achieve 3 log-reductions is reduced from to 1.25 min to 0.6 min. Consequently, the addition of the nanoemulsified *D-*limonene is also effective at reducing the intensity of the thermal treatment also under non-isothermal conditions.

## Discussion

Food producers will face several challenges during the next decades. It is expected that the global population will be higher than 9 billion by 2050^41^. On top of that, a growing middle class implies an increase in the demand of high quality products, with fresh-like attributes (sensorial and nutritional)^16^. These challenges require the development of novel technologies for food processing, preservation and stabilization^1^. In this sense, the application of nanotechnology to enhance the antimicrobial effect of emulsified natural antimicrobials seems a promising technique for microbial inactivation.

The first studies analyzing the effect of natural antimicrobials in the heat resistance of various vegetative cells observed a mild impact (on the order of a three-fold reduction)^42–45^. However, when *D-*limonene was present in form of nanoemulsion, Maté *et al*.^26^ observed a 100-fold reduction in the thermal resistance of *L. monocytogenes* in Tryptic Soy Broth. A dramatic decrease in heat resistance compared to the one observed in previous research works, where the antimicrobial was not nanoemulsified. A similar effect on the heat resistance of *L. monocytogenes* was also observed in apple juice, demonstrating its potential application in foods^25^. In a similar study for *Salmonella* Senftenberg^27^, a reduction in the microbial resistance by a factor of 50 was observed. In this study we have observed a reduction in the D-value by a factor of 25. The lower impact observed in our study can be associated to the effect of the heating medium, whose composition can have a strong impact on the effectiveness of the nanoemulsified antimicrobial^25^. It could also be associated to the emulsifier used, soya lecithin, with a higher molecular weight than Tween 80, that could interfere with the interaction between the bacterial membrane and the antimicrobial. Soya lecithin has also been shown to promote only slightly the aqueous-phase concentration of essential oils, in contrast to other emulsifiers, which, in consequence, significantly enhanced the bactericidal activity of these same essential oils^46^. Even the emulsification procedure could have an effect, catastrophic phase inversion versus high shear homogenisation, either of which may diminish the antimicrobial effectiveness of the nanoemulsion. Indeed, our data showed a loss on the bactericidal effect of the nanoemulsion when soya lecithin was used instead of Tween 80 and propylene glycol. Still, these ingredients could be more appreciated by potential customers, due to their preferences for more natural food products^47^. Despite a lower effect on the thermal resistance of the cells, our results imply that the intensity of the isothermal treatment could be reduced considerably without affecting the safety of the product with respect to *L. monocytogenes*. Considering the negative impact of the thermal treatment in the quality of most food products, the reduction in the treatment intensity enabled by the nanoemulsified antimicrobial would improve the product quality. These data open the door for new researches that help to improve this natural based technology with the aim of achieving or even overpassing the effect of the traditional ones.

The shaded areas of Fig. 2 depict the prediction intervals of the inactivation model based on the parameters estimated from isothermal experiments. In every case, there are relatively large deviations between the model predictions based on isothermal data and the dynamic observations. This is a common observation in microbial inactivation^30–35,48^. It has been reported that *L. monocytogenes* can develop a physiological response to mild stresses, that increases its resistance to posterior treatments^36,37^. This adaptive response has also been observed for microbial cells which had been previously received shocks (treatments of short duration and high intensity)^49,50^. This physiological response can also be relevant during non-isothermal treatments, when the temperature is raised slowly and enables cells to develop a response that increases its resistance. This type of response is usually called “stress acclimation”. Garre *et al*.^33^ observed that the adaptive response of *L. monocytogenes* cells subjected to non-isothermal treatments was irrelevant for heating rates higher than 3 °C/min. However, stress acclimation could increase the D-value by a factor of 2.2 for lower heating rates. Those results are consistent with the results obtained in this investigation for a heating rate of 1 °C/min (Fig. 2A).

It has also been reported that high heating rates can also have an impact on the microbial response of microorganisms. Several studies have shown that heating rates above 10 °C/min can reduce the resistance of microbial cells to thermal stress^35,51^. This effect results in a lower microbial count to the one predicted based on isothermal experiments. The results obtained in this investigation for a heating rate 20 °C/min are consistent with this hypothesis. Regarding the results obtained for a heating rate of 10 °C/min, the samples without limonene show an additional sensitivity of microbial cells, which is consistent with the hypothesis that high heating rates increases the effect of the thermal treatment. However, the response of samples supplemented with nanoemulsified limonene is more complex. At the beginning of the treatment, a high reduction in the microbial load is observed, compatible with the hypothesis that high heating rates result in further microbial inactivation. However, at the end of the treatment (*t* = 2 min), the microbial count is higher than predicted based on isothermal experiments. This could be explained by a physiological response of the cells to the treatment that increases its thermal resistance. In many cases, this response can increase the ability of cells to survive stresses of a different nature than the one that caused the response^52^. In a similar fashion, the addition of the nanoemulsified antimicrobial could trigger the physiological response that increases its thermal resistance, resulting in survivor curves similar to those observed when stress acclimation is relevant.

It is worth highlighting that our experimental results have been obtained using laboratory media (BPW), not an actual food product. Most food products are complex media with several components (e.g. fat and fibers) that can affect microbial growth and inactivation^25,53–56^. For that reason, exploratory research works aiming to understand the microbial response, rather than to validate its application in an actual food product are usually performed using standardized laboratory media. Naturally, before being applied in an actual food process, the use of nanoemulsified D-limonene to improve the efficiency of dynamic thermal treatments should be tested on actual food products. Nevertheless, our experimental results, albeit obtained in laboratory media, point out that dynamic effects can be very relevant and that they should be analysed when this type of combined treatment is validated in an actual food product.

There are still several technological limitations to overcome before antimicrobials can be used at large scale in food production. Nevertheless, our results show the big promise that natural antimicrobials bring to reduce the intensity of pasteurization processes. The application of nanotechnology to produce a nanoemulsion based on a natural essential oil emulsified using an also natural substance can solve several of these shortcomings, increasing the antimicrobial effect of the essential oil, as well as increasing its stability. Our results confirm previous investigations, proving the strong effect of nanoemulsified *D-*limonene on the thermal resistance of *L. monocytogenes*. We also show that the design of non-isothermal treatments bring additional complexity, due to several physiological responses (e.g. stress acclimation) that are not relevant in isothermal treatments. In this sense, the application of the antimicrobial may contribute to triggering the adaptive response of the microbial cells, reducing the effectiveness of the treatment if applied at conditions that enable for this behaviour (*i.e*. a slow heating rate). But overall, our results show the positive impact on food safety of the synergistic effect observed after the combination of a nanoemulsified antimicrobial with heat, moreover, when the target microorganism is a foodborne pathogen of great present concern, such as *L. monocytogenes*. Still, considering that heat treatments applied in industry are non-isothermal, further research is needed in order to define realistic treatments supplemented by a nanoemulsified *D-*limonene.

## Methods

### Bacterial strain

*L. monocytogenes* CECT 4032 was used in this study and it was provided by the Spanish Type Culture Collection (CECT, Valencia, Spain). This strain was stored at −80.0 °C (30% glycerol) until use. Fresh cultures of *L. monocytogenes* were prepared by inoculating a loop of the cryopreserved culture in tryptic soy broth (TSB; Scharlau Chemie S.A., Barcelona, Spain) and incubating overnight at 37 °C until the stationary growth phase was reached.

### Preparation of nanoemulsions

Nanoemulsions of *D-*limonene were prepared following the protocol described by Maté *et al*.^26^, but with some significant modifications. Aqueous phase was prepared by mixing 20 mL of sterile distilled water and 10 g of commercial soya lecithin (Korott, Alicante, Spain) and keeping the mixture for 1 h at 50.0 °C in a water bath to allow for lecithin hydration. After hydration, the mixture was homogenised with a mixer. Once the aqueous phase was ready, 3.23 mL of *D-*limonene (Sigma Aldrich Chemie, Steinheim, Germany) were added to 2.5 g of the aqueous phase. Then, sterile distilled water was added to get a final volume of 20 mL. Nanoemulsion was prepared using an ultra-turrax ultrasonic homogeniser at 300–500 rpm for 15 min, under aseptic conditions. Final concentration of *D-*limonene in the nanoemulsion was 1M. Nanoemulsions were aliquoted in pre-sterilized test tubes and stored in refrigeration until use. Droplet size was determined at the beginning and at the end of the experiment. Size distribution of the oil droplets were determined by the laser light scattering method using Mastersizer 2000 (Malvern Instruments, Worcestershire, UK), as already described^26^. No differences were found in size distribution along the time the present research was performed (data not shown). Droplet size distribution was similar to those obtained in previous researches for *D*-limonene nanoemulsions^20,25^.

### Heat treatments

Thermal inactivation kinetics for *L. monocytogenes* in buffered peptone water (BPW; Scharlau Chemie) supplemented or not with 0.5 mM nanoemulsified *D*-limonene was determined in a thermoresistometer Mastia as described by Conesa *et al*.^57^. *D*-limonene was added to pre-sterilized BPW in sterile conditions. Then, the vessel of the thermoresistometer was filled with 400 mL of pre-sterilized BPW supplemented (or not) with *D*-limonene. Heat treatments were conducted at 50.0, 52.5, 55.0 and 57.5 °C for isothermal treatments. For dynamic experiments, initial temperature was set at 40 °C. Then, heating rates of 1, 10 or 20 °C/min were applied. Once the heating medium temperature had attained stability (±0.05 °C), it was inoculated with 0.2 mL of the cell culture (approx. 10^9^ cells mL^−1^). Every experiment was made with stirring in the vessel. At preset intervals, 1 mL samples were collected into sterile test tubes, which were cooled down in ice to stop the thermal treatment. Then, decimal dilutions were immediately performed. Surviving cells were enumerated in tryptic soy agar (TSA, Scharlau Chemie). Plates were incubated for 24 h at 37 °C. Each treatment was assayed by triplicate in independent experiments performed in different days.

### Mathematical modelling and data analysis

Microbial inactivation was described using the Geeraerd model for microbial inactivation^58^. This model is an extension of the 1^st^ order kinetics model which introduces to coefficients (*α* and *β*) to describe sigmoid survivor curves. It can be written as a differential equation as shown in Eq. (1), where *N(t)* is the microbial count at treatment time, *t*.

1 $$\frac{dN}{dt}=\alpha \cdot {k}_{\max }\cdot \beta \cdot N(t)$$

The coefficient *α* describes shoulder effects (deviations from linearity in survival curves that take place at the beginning of the thermal treatment), whereas *β* describes tails (deviations at the end of the treatment). None of the survivor curves obtained show any shoulder effect, so *α* was fixed to one in every case. Consequently, this coefficient was set to one, so it does not have any impact. The equation used in the Geeraerd model for *β* is shown in Eq. (2), where *N*_*tail*_ is the tail height. This parameter represents an asymptote of the survivor curve. Experiments without the addition of limonene did not have any tail. For that reason, parameter *β* was fixed to one in those cases. On the other hand, survivor curves where limonene was added to the heating media did have tails.

2 $$\beta =1-\frac{{N}_{tail}}{N}$$

Both *α* and *β* take values between zero and one, so *k*_*max*_ represents the maximum inactivation rate. Instead of *k*_*max*_, in this article we report the D-value (${D}_{T}=\frac{\mathrm{ln}\,10}{{k}_{max}}$), which is frequently used in the field of food microbiology. This parameter represents the time that a heat stress must be held to reduce the microbial load a 90% (without considering non-linearities). The rate of microbial inactivation has a strong dependence with temperature. This relationship has been modelled using the Bigelow model^59^, which assumes a log-linear relationship between the D-value and temperature. This is shown in Eq. 3, where *z* is the z-value of the microorganism, which defines the temperature increase required for a ten-fold reduction of the D-value. This model introduces a reference temperature (*T*_*ref*_), without any biological meaning, but with a positive impact on parameter identifiability^60^. The parameter *D*_*ref*_ is the estimated D-value at *T*_*ref*_.

3 $${\log }_{10}\,D(T)={\log }_{10}\,{D}_{ref}-\frac{T-{T}_{ref}}{z}$$

For isothermal conditions (and in the absence of shoulders), the Geeraerd model has the analytical solution shown in Eq. (4). Parameter estimates (for *N*_0_, *N*_*tail*_ and *D*) have been calculated from isothermal experiments by fitting this equation to the experimental data. The experiments were estimated using the one-step fitting algorithm, where model parameters for every treatment temperature are estimated in one step. This algorithm has proved more accurate than the two-step algorithm, where parameter are estimated sequentially^61–63^. It has been fitted using the Levenberg-Marquard algorithm, implemented in the *minpack.lm* R package^64^. These parameter values have been used to predict the microbial response under non-isothermal conditions using the *bioinactivation* R package^65,66^. Prediction intervals for the microbial response under non-isothermal conditions have been estimated using Monte Carlo simulations^65,67^, considering that each model parameter follows a normal distribution with the mean and standard deviations estimated from the isothermal experiments. The quantiles of 1000 Monte Caro simulations were used to calculate the prediction interval. All the calculations were performed using the R programming language version 3.5.3^68^.

4 $$N(t)=({N}_{0}-{N}_{tail}){e}^{-\frac{\mathrm{ln}10}{D}t}+{N}_{tail}$$

## Data Availability

The datasets generated during and/or analysed during the current study are available from the corresponding author on reasonable request.
